# Defect Classification of Green Plums Based on Deep Learning

**DOI:** 10.3390/s20236993

**Published:** 2020-12-07

**Authors:** Haiyan Zhou, Zilong Zhuang, Ying Liu, Yang Liu, Xiao Zhang

**Affiliations:** College of Mechanical and Electronic Engineering, Nanjing Forestry University, Nanjing 210037, China; zhouhaiyanzj@njfu.edu.cn (H.Z.); zzl0702@njfu.edu.cn (Z.Z.); lyang_03@njfu.edu.cn (Y.L.); zx_xhx@njfu.edu.cn (X.Z.)

**Keywords:** green plum, defects, deep learning, classification

## Abstract

The green plum is rich in amino acids, lipids, inorganic salts, vitamins, and trace elements. It has high nutritional value and medicinal value and is very popular among Chinese people. However, green plums are susceptible to collisions and pests during growth, picking, storage, and transportation, causing surface defects, affecting the quality of green plums and their products and reducing their economic value. In China, defect detection and grading of green plum products are still performed manually. Traditional manual classification has low accuracy and high cost, which is far from meeting the production needs of green plum products. In order to improve the economic value of green plums and their products and improve the automation and intelligence level of the product production process, this study adopted deep learning methods based on a convolutional neural network and cost-effective computer vision technology to achieve efficient classification of green plum defects. First, a camera and LEDs were used to collect 1240 green plum images of RGB, and the green plum experimental classification standard was formulated and divided into five categories, namely, rot, spot, scar, crack, and normal. Images were randomly divided into a training set and test set, and the number of images of the training set was expanded. Then, the stochastic weight averaging (SWA) optimizer and w-softmax loss function were used to improve the VGG network, which was trained and tested to generate a green plum defect detection network model. The average recognition accuracy of green plum defects was 93.8%, the test time for each picture was 84.69 ms, the recognition rate of decay defect was 99.25%, and the recognition rate of normal green plum was 95.65%. The results were compared with the source VGG network, resnet18 network, and green lemon network. The results show that for the classification of green plum defects, the recognition accuracy of the green plum defect detection network increased by 9.8% and 16.6%, and the test speed is increased by 1.87 and 6.21 ms, respectively, which has certain advantages.

## 1. Introduction

Green plums are widely distributed in hills and sloping forests all over China. Green plums are grown not only in China but also in Vietnam, Thailand, the Philippines, Indonesia, and other countries surrounding China. The green plum is sweet in taste and flat with a thin skin, thick and juicy flesh, small nucleus, crisp texture, high acidity, high fruit acid and vitamin C content, and high nutritional value [1]. The green plum contains a variety of natural acids such as citric acid, which is indispensable for human metabolism [2]. It is a rare alkaline fruit that also contains threonine and other amino acids and flavonoids, which are extremely conducive to the normal progress of the body’s protein composition and metabolic function and have obvious preventive and curative effects on ubiquitous cardiovascular, urinary, and digestive diseases. Green plums are a popular kind of dual-purpose medicinal and food resource with multiple health care functions [3].

However, green plums are susceptible to collisions, pests, and diseases during growth, picking, storage, and transportation. Surface defects (such as rot, scars, cracks, rain spots) of green plums affect the quality of green plums and their products and reduce their economic value. The classification of the four main defects of green plums (rot, scars, cracks, rain spots) is conducive to the manufacturing of different green plum products, such as preserves, extracts, and wine [4]. This classification process also aids efficiency and reduces waste. In China, the sorting of green plums is mostly carried out through manual operation, which has disadvantages such as low efficiency, low accuracy, and high cost. Therefore, in order to increase the economic added value of green plums and their products, it is of great significance to sort green plums according to multiple types of defects and realize an automatic detection and classification system.

As daily life improves, customers pay more attention to fruit quality. Nondestructive detection and processing techniques have been developed rapidly for fruit inspection [5,6]. Many studies focused on measurement of fruit quality and defects using multi-spectral or hyperspectral imaging technologies [7,8]. Kleynen et al. [9] developed a visible/near-infrared band multispectral vision system, using related pattern matching algorithms and defect segmentation based on Bayes’ theorem. According to the actual intact rate of 90%, fewer than 2% of defective apples were classified as intact apples. In the study of Blasco et al. [10], based on the multispectral data and morphological characteristics, 11 kinds of defects (chilling injury, *Penicillium digitatum*, scales, medfly, anthracnose, stem-end injury, scarring, thrips scarring, sooty mold, phytotoxicity, oleocellosis) were identified and classified on the surfaces of citrus fruit. More than 2000 kinds of citrus fruits were identified, and the correct rate reached 86%. Huang et al. [11] used a new non-contact multi-channel spectroscopy system for non-destructive testing of internal defects in apples and established a classification model based on partial least squares discriminant analysis (PLSDA). The overall accuracy of the three detection directions of the stem end towards the light source, the calyx end towards the light source, and the stem calyx axis perpendicular to the light source reached 91.5%, 89.2%, and 93.1%, respectively. Based on visible/near-infrared (Vis-NIR) hyperspectral imaging technology, Zhang et al. [12] produced a multi-spectral image classification algorithm. Using Nanfeng citrus fruits as the test material, four types of anthracnose, scarring, decay, and thrips scarring were detected. The defects were classified, and the classification accuracy rate was 96.63%. The high price and specific working band of hyperspectral equipment limit its use in the fruit sorting production line.

In recent years, with the development of machine vision, domestic and foreign scholars have widely applied nondestructive machine vision testing to the identification and classification of agricultural products [13]. Based on texture uniformity measurement technology, Hassan and Nashat [14] achieved a 100% recognition rate of healthy olives. The recognition rate of olives with small defects reached 99%, and the recognition rate of olives with large defects reached 98%. Capizzi et al. [15] used a radial basis probabilistic neural network (RBPNN) to classify the color and texture of citrus surface defects. By calculating the gray level co-occurrence matrix, the texture and gray level features of the defect area were extracted. The five categories of morphological defects, slight color defects, black mold, and good fruits were classified, with a total error rate of 2.75%. Based on computer vision, Yogesh et al. [16] extracted the characteristics of fruit hardness, size, contour, and texture and used a support vector machine (SVM) classifier to classify defects of apples, pears, and pomegranates with accuracy rates of 98.5%, 96.6%, and 92.5%, respectively. Sujatha et al. [17] used the histogram of oriented gradients (HOG) feature extraction method and the bagged decision tree (BDT) classification method to classify apples into healthy and defective categories, with an accuracy rate of 96%. Bhargava and Barisal [18] compared the classification quality of four different classifiers, k-nearest neighbor (k-NN), support vector machine (SVM), sparse representative classifier (SRC), and artificial neural network (ANN), on four fruits: apples, bananas, oranges, and avocados. The system performance was verified by k-fold cross-validation technology. When k = 10, the maximum accuracy of fruit detection was 80.00% (k-NN), 85.51% (SRC), 91.03% (ANN), and 98.48% (SVM).

The traditional machine learning method needs to extract features manually, which can easily cause related features to be incomplete, thereby reducing the accuracy of recognition. In recent years, with the development of computer science, the combination of machine vision and deep learning technology has gradually been applied to the field of fruit quality sorting. Fernando Villacres and Auat Cheein [19] developed, tested, and evaluated a method based on deep learning using a portable artificial vision system to improve cherry harvest with an accuracy rate of 85%. Wang and Chen [20] applied an improved eight-layer convolutional neural network (CNN) to the classification of 18 kinds of fruits including Anjou pears, blackberries, black grapes, blueberries, Bosch pears, cantaloupes, and watermelons with an accuracy rate of 95.67%. Wan et al. [21] applied the improved Faster R-CNN to the recognition of apples, mangos, and oranges, and the average recognition rate reached about 91%. da Costa et al. [22] applied ResNet50 to the recognition of tomato external defects, and the recognition rate reached 94.6%. Zhou et al. [23] analyzed the potential of deep learning as an advanced data mining tool in food sensory and consumption research. Their survey results show that deep learning is superior to manual feature extraction and traditional machine learning algorithms, and deep learning can be used as a promising tool for food quality and safety inspection [24].

The CNN automatically extracts features from the image sample dataset through the convolutional layer, compresses the input feature map in the pooling layer to simplify the network calculation complexity, and extracts the main features, and finally all the connected feature output values are sent to the classifier for feature classification in the fully connected layer. This method overcomes the limitations of manual feature extraction. In this study, the improved VGG convolutional neural network was applied to the machine vision system to realize detection and classification of multi-type defects on the surfaces of green plums, improve the accuracy and speed of green plum defect detection, and provide technology support for the automation of green plum processing.

The contributions of this paper are (a) multi-defect classification for green plums; (b) the use of a convolutional neural network in the multiple defect classification and recognition of green plums; and (c) the application of a stochastic weight averaging (SWA) optimizer and w-softmax loss function in the green plum defect detection network based on a deep learning network, to enable the network to accurately learn green plum features, avoid premature convergence of the network, and effectively improve the classification performance of the network.

## 2. Materials and Methods

### 2.1. Imaging

**Figure 1:**
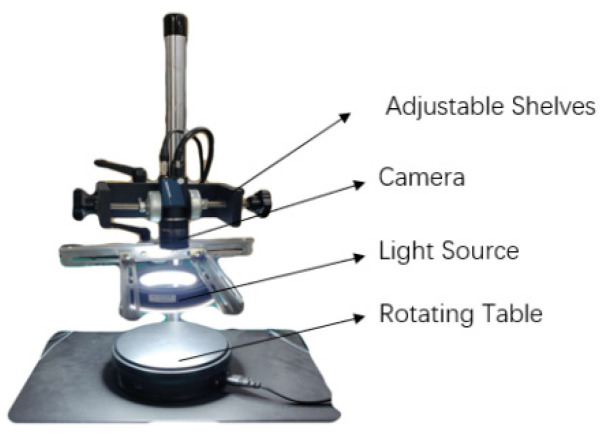
Image acquisition device.

In this study, a batch of green plums in Dali, Yunnan Province, were purchased from the Internet and used in this research, and 1240 green plum RGB images were collected by the visible image acquisition system (Figure 1).

The optical lens of the image acquisition system used the M1620-MP2 industrial camera lens produced by Japan Computar, with a focal length of 16 mm, a minimum object distance of 20 cm, and 5 million pixels. A MER-531-20GC-P industrial camera from Daheng Image Technology Co., Ltd, Beijing, China. was used, and the camera had an OnsemiPYTHON5000 frame exposure Complementary Metal Oxide Semiconductor (CMOS) sensor chip, an integrated Gigabit Ethernet interface, and color spectrum, suitable for a relatively harsh working environment. The light source was an LED ring light source.

### 2.2. Preprocessing Images

**Figure 2:**
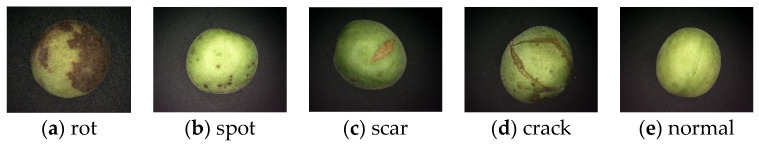
The original image collected by the system.

**Figure 3:**
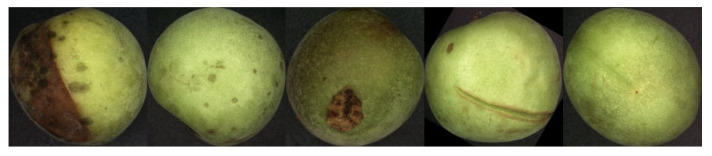
Preprocessed image.

The pictures collected by the camera were 2592 × 2048 pixels, and examples of the original images obtained are shown in Figure 2. In order to reduce the influence of the background on the identification of green plum defects, background cropping was performed. Oversized images result in lower quality image analysis and reduced processing speed. In order to achieve a faster processing speed without distorting the green plum image, image preprocessing was required. The pre-processing process was as follows: The 11 × 11 convolution kernel was used for Gaussian filtering to convert the image into grayscale, the adaptive threshold was used for binarization, the Laplacian operator was used for filtering, and the Canny operator was used for edge extraction to obtain the smallest outer rectangle of the edge. The image size was cut and finally adjusted to 100 × 100 pixels for experimentation, as shown in Figure 3.

### 2.3. Dataset

The collected images were divided into five categories in this study: rot, cracks, scars, rain spots, and intact skin, as shown in Figure 2. Due to the diversity of fruit surface defects, there are multiple defects in the skin. The classification of green plums in this study was based on injury degree; the severity of decay is the highest, followed by cracks, scars, and rain spots. For example, the green plum on an image had both cracks and rot, so it was classified as rot. The 1240 images collected were divided into 5 categories, and each category was randomly selected at a ratio of 4:1 to divide the images into a training set and a validation set. In the process of expanding the dataset, the data of the training set and the test set were mirrored, the data were increased at every 45° rotation angle, and a total of 10 times increments were obtained. The specific data are shown in Table 1.

### 2.4. Neural Network Architecture

The VGG network has good classification performance [25]. The green plum defect detection network in this study was based on the VGG network architecture. Combined with the classification feature requirements of the green plum data, the network structure was finely adjusted using w-softmax loss function and a SWA optimizer. Finally, through transfer learning, the weights of the model trained on ImageNet were transferred to the finely adjusted network architecture for classification training of green plum defects.

#### 2.4.1. Green Plum Defect Detection Network Structure

**Figure 4:**
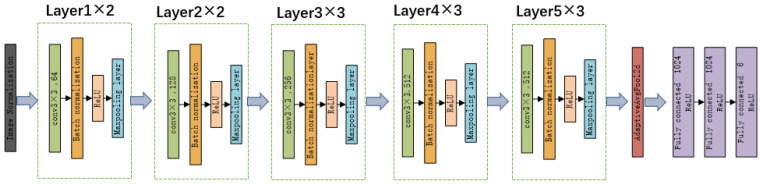
Green plum defect detection network structure.

The green plum defect detection network consists of an input layer, a convolution layer, a pooling layer, a fully connected layer, and an output layer. The entire network has 16 layers [26]. The structure of the green plum defect detection network is shown in Figure 4. The network has a total of 16 layers. The convolutional layer has 13 layers. Each convolution layer is composed of convolution layer, batch normalization (BN), the Rectified Linear Unit (ReLU) activation function, and the maxpooling layer. The fully connected layer has 3 layers. There is an adaptive pooling function (AdaptiveAvgPool2d) between the convolutional layer and the fully connected layer.

The convolution operation is used to extract features [27,28]. The network used all 3 × 3 convolution kernels for convolution. A smaller convolution kernel can reduce network parameters, reduce the amount of calculation, and increase the calculation speed. The data input can be normalized in batch normalization (BN) to reduce the influence of offset and increase of input data. ReLU was used as the activation function, making the output of some neurons 0, which causes the sparsity of the network, reduces the interdependence of parameters, and alleviates the occurrence of overfitting.

Maxpooling was used to extract several eigenvalues from a filter. Only the largest pooling layer was obtained as the reserved value, and all other feature values were discarded. The largest value means that only the strongest of these features is retained, and other weaker features are discarded. The purpose of pooling was to ensure the invariance of the position and rotation of the features and perform dimensionality reduction operations on the features of the filter layer, reduce the size of the feature map, increase the receptive field, reduce the number of model parameters, and reduce over-fitting problems. This process is beneficial to deepening and widening the network [21,29].

Using the adaptive pooling function, the system can adaptively adjust the size and stride of the convolution kernel for input information, and the output can reach the required size for any pixel size of the input.

The FC layer has the largest number of parameters in the entire network and acts as a classifier in the entire convolution neural network [30]. It plays the role of mapping the learned distributed feature representation to the sample label space, the weighted sum of the source vector, which is similar to the target vector. Fewer units can reduce the parameter storage and training time of CNNs.

#### 2.4.2. SWA Optimizer

The optimizer manages and updates the values of learnable parameters in the model so that the model output is closer to the true label. This network used the stochastic weight averaging (SWA) [31] optimizer, which used a periodic learning rate instead of a constant learning rate. When the learning rate changes, the SWA optimizer adds a moving average to the stochastic gradient descent (SGD) optimizer to limit the change of its weight. The local minimum generated at the end of each learning rate cycle tends to accumulate in the edge area of the loss surface. The loss value on these edge areas is small. By averaging several such points, it is possible to obtain an even lower rate of loss and a globalized general solution. Thus, a periodic moving average is created. The period is c; that is, a moving average is performed every c steps. SWA takes the sliding average of the parameters in the period and limits the update frequency, which can reduce the weight oscillation problem in the reverse process.

The learning rate reflects the magnitude of the parameter updating. If the learning rate is too large, the parameters to be optimized fluctuate near the minimum value and do not converge. If the learning rate is too small, the parameters to be optimized converge slowly. Using a periodic constant learning rate, in each cycle, linear iteration was used to reduce the learning rate, as shown in Formula (1) [31]:

(1) $$\alpha (i)=(1-t(i))\alpha_{1}+t(i)\alpha_{2},$$

(2) $$t(i)=\frac{1}{c}(\mathrm{mod}(i-1,c)+1).$$

The average w for each learning rate period is

(3) $$w_{swa}\leftarrow \frac{w_{swa}\cdot n_{\mathrm{mod}els}+w}{n_{\mathrm{mod}els}+1}.$$

At the end of each learning rate period, the weight of the second model was used to update the weight of the first model, as shown in Equation (3). Therefore, in the training phase, only one model needed to be trained, and two models were stored in memory. Only the averaging model was required for forecasting. This method of prediction is much faster than the previously described integration.

#### 2.4.3. W-Softmax Loss Function

In the feature classification subtask, the FC layer with softmax loss is the mainstream, and the design of the loss function plays an important role in the training of the deep network. Commonly used classification loss functions include the mean square error loss function and the cross entropy loss function. In this study, the output of the model was passed through the software function to obtain the probability distribution of the output classification and then compared with the standard answer. The cross entropy was calculated, and the loss function was obtained. The softmax function is used in the multi-classification process. It maps the output of multiple neurons to the (0,1) interval, which can be understood as a probability for multi-classification. w-softmax (a negative focused weights-biased softmax) [32,33] increases the training loss by increasing the negative weight value, increasing the value of γ to change the decision boundary, increasing the category variance, reducing the variance of the same category, and improving the discrimination rate of features, thereby improving classification accuracy. Like the traditional softmax loss method, w-softmax has high computational efficiency in training and optimization. Softmax loss is a special case of w-softmax loss, namely, γ=0. The norm L_2_ was used to normalize the weight vector of all classes, and then the weight vector of each negative class was evaluated using the following formula [34,35]:

(4) $$w_{i}^{\prime }=\frac{\gamma w_{c}+w_{i}}{∥\gamma w_{c}+w_{i}∥},(i\neq c),$$

where c is the index of the positive classifier weight vector, i is the index of the negative classifier weight vector, $w_{c}$ is the positive classifier weight vector, and $w_{i}$ is the negative classifier weight vector.

The weight matrix $w_{c}$ is as follows:

(5) $$w_{c}={\left[\begin{array}{cc} \sqrt{\frac{{\left(c-1\right)}^{2}-1}{{\left(c-1\right)}^{2}}}w_{c-1} & 0\\ -\frac{1}{c-1} & 1 \end{array}\right]}_{(c-1)\times c},c>2.$$

The function of w-softmax meets the probability distribution requirements of the n output of the n classification. In the c classification, for the input feature x to the last FC layer with the label c, the following formula was used to evaluate its correct probability $p_{c}$ and error probability $p_{i}$ (i≠c), where $\theta_{c}$ and $\theta_{i}^{\prime }$ represent the angle between the weight vector $w_{c}$ and the feature x and the angle between $w_{i}^{\prime }$ and x.

(6) $$p_{c}=\frac{\exp(∥x∥\cos\theta_{c})}{\exp(∥x∥\cos\theta_{c})+\sum_{j\neq c}^{C}\exp(∥x∥\cos\theta_{j}^{\prime })},$$

(7) $$p_{i}=\frac{\exp(∥x∥\cos\theta_{c})}{\exp(∥x∥\cos\theta_{c})+\sum_{j\neq c}^{C}\exp(∥x∥\cos\theta_{j}^{\prime })}.$$

The cross entropy loss function was obtained using w-softmax:

(8) $$L=-\log\frac{\exp(w_{c}^{T}x)}{\sum_{j=1}^{C}\exp(\frac{\lambda w_{c}^{T}+w_{j}^{T}}{∥\lambda w_{c}+w_{j}∥}x)}.$$

## 3. Results

All the codes of the green plum defect detection network were written in Python, using the deep learning framework PyTorch to define the network calculation graph, and optimized based on the transfer learning strategy to accelerate the training and learning speed. The software, hardware, and compilation environment configuration of this experiment is shown in Table 2:

The green plum defect detection network training parameter settings were as follows: Batchsize was set to 32, the number of SWA-averaged periods was 10, the initial learning rate was 0.1, the weight decay rate was 1e-4, and the SWA learning rate was 0.05. The green plum training set data were imported to train the network until the minimum loss was obtained. The loss value remained stable at 20 epochs, and the training of the green plum defect detection network model was completed.

The test set of green plum data was imported into the trained green plum defect detection network model, and the test results were obtained. From Table 3, it can be seen that the average precision value of the green plum defect detection network for the surface defect recognition and classification of green plums reached 93.8%, and the test time of a single green plum was 84.69 ms. The recognition rate of the green plum defect detection network was rot > normal > rain spot > scar > crack. The decay feature was the most obvious, with the highest recognition rate of 99.25%, followed by non-defective green plums with 95.65%, the rain spot recognition rate of 93%, and the poor recognition of scars at 84.29% and cracks at 78.13%.

**Figure 5:**
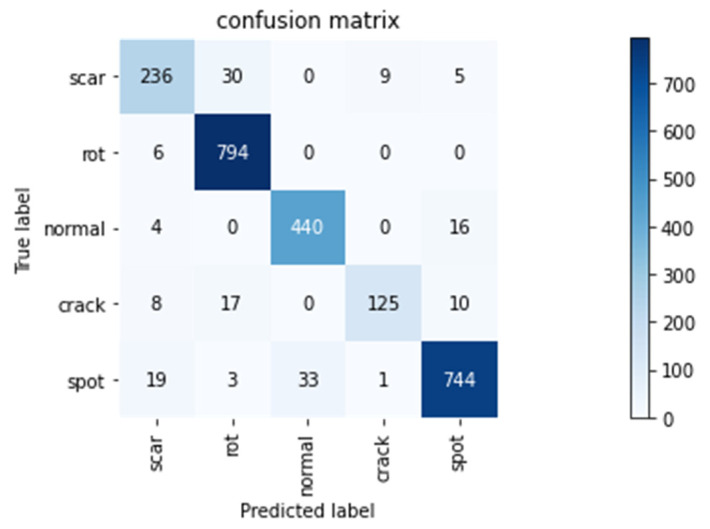
Confusion matrix diagram of green plum defect detection network test.

The confusion matrix in Figure 5 obtained by the green plum defect detection network shows that out of 280 scar defect green plum images, 236 were correctly identified, 30 were mistaken for rot, 9 were mistaken for cracks, and 5 were mistaken for rain spots; among the rot defective green plum images, 794 were correctly identified, and 6 were misidentified as scars; 440 of the 460 normal green plum images were recognized correctly, 4 were misidentified as scars, and 16 were misidentified as rain spots; 125 of 160 crack defects were correctly identified, 8 were mistakenly identified as scars, 17 were mistakenly identified as rot, and 10 were mistakenly identified as rain spots; 744 out of 800 rain-like defects were correctly identified, 19 were mistaken for scars, 3 were mistaken for decay, and 33 were mistaken for normal.

**Figure 6:**
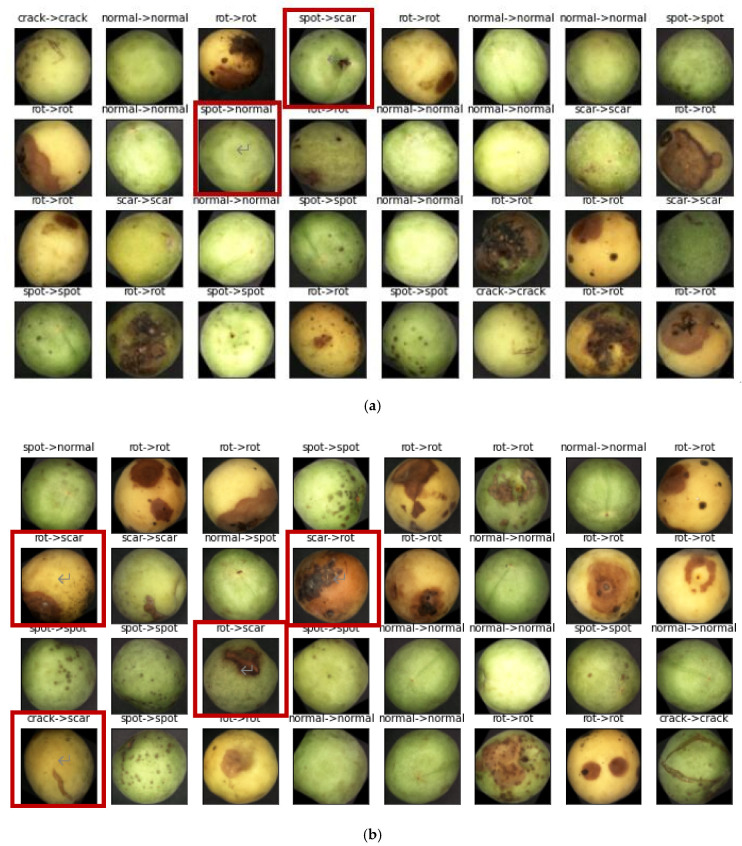
Results of the test (**a**) test 1 (the red box is the confusion of spot); (**b**) test 2 (the red box is the confusion of scars, crack and rot).

It can be seen from the confusion matrix in Figure 5 that rain spots were misjudged as scar defects, and the proportion of misclassifications as normal was relatively high, accounting for 2.4% and 4.1%, respectively. Screenshots of the identification results and analysis are shown in Figure 6 below. The red boxes represent misrecognized images, and the rain spot defect in frame no. 1 in Figure 6a was mistakenly identified as a scar. The main reason is that there are fruit stems in this picture, and the network mistook the fruit stems as scars. The features of scars are more obvious than rain spots, so it was misjudged. The rain spot defect in frame no. 2 was identified as a normal green plum. The rain spot on the picture is relatively off-center, and the small number and shallow depth of the rain spots led to them not being recognized.

It can be seen from Figure 5 of the confusion matrix that scars and decay are easy to confuse. Scar defects misjudged as decay accounted for 10.7%. In Figure 6b, the red box is the confusion of scars and decay. The shape of scars and decay is irregular, and the colors are similar but common, though the scar is darker. In Figure 6b, the rot in box 1 was misjudged as a scar, and the color of the rot is darker, which is prone to misjudgment; the scar in box 2 was misjudged as rot, which may interfere with the color of the green plum itself; and the rot in box 3 was misjudged as a scar. The green plum image was in a state of coexistence between scars and rot. The outer circle of the defect was scarred, but the inner circle of the defect was in a rotten state, causing a misjudgment; the crack in box 4 was misjudged as a scar, and the crack in the picture was not deep enough, failing to show the characteristics of cracks, causing misjudgment.

## 4. Discussion

The VGG network has good classification performance, and the green plum defect detection network was improved on the basis of the VGG network. The green plum data were imported into the source VGG network for testing. The moment was set to 0.9, the learning rate was set to 1e-4, and the batch size was set to 32. The VGG network was then compared with the green plum defect detection network in terms of loss curve and test results.

**Figure 7:**
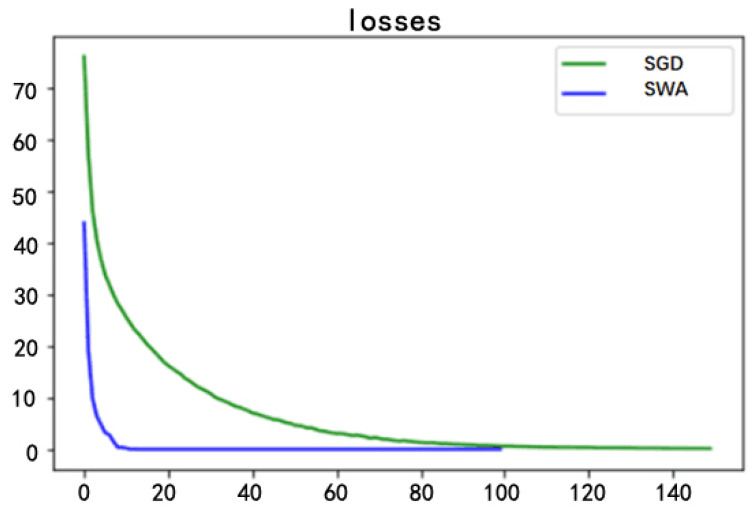
Loss curve.

The green plum defect detection network used the SWA optimizer, while the VGG network used the SGD optimizer. As shown in Figure 7 below, the VGG network training needed to be iterated for 140 epochs to reach the minimum loss, while the loss curve of the green plum defect detection network after 100 epochs of training iterations converged, reached the minimum loss, and obtained the fitting state. In the training process, the loss curve of the VGG network converged slowly and required more training time. The green plum defect detection network converged faster and reached a stable state. This shows that the SWA optimizer can achieve a good convergence effect, and the convergence speed is fast and stable. The green plum defect detection network performed better than the VGG network in the identification and classification of green plum defects. The loss value decreased faster during the training process, and the convergence requirement was reached faster.

From the test results shown in Table 4, it can be seen that the green plum defect detection network can identify the main features of each defect and distinguish them according to the main features. Not all CNN networks have a high recognition rate after training. Incorrectly identifying the main features produces misjudgments. The network structure and network parameters of the CNN change the recognition ability of main features. The green plum defect detection network was optimized on the basis of the VGG network. It can be seen from Table 4 that the average precision value of the green plum defect detection network for the identification and classification of green plum surface defects reached 93.8%, while the VGG network only reached 84%. The green plum defect detection network improved the recognition accuracy of green plum rot defects, rain spot defects, scar defects, crack defects, and normal green plums. The identification of crack defects increased from 55.63% of the VGG network to 78.13%, the highest increase. The green plum defect detection network achieved good results in the recognition of decay defects, reaching 99.25% accuracy, and the recognition rate of normal green plums reached 95.65%. The results show that using the w-softmax loss function can help CNNs learn more distinguishing features, increase the gap between categories, reduce the gap between the same categories, improve the discrimination rate of features, and thus improve the classification accuracy. The test time of each picture of the green plum defect detection network was 84.69 ms, and the test time of each picture of the VGG network was 86.56 ms because the number of neurons in the fully connected layer in the green plum defect detection network is 1024, and reducing the number of parameters of the fully connected layer can shorten the test time and increase the test speed.

Jahanbakhshi used a self-designed CNN network [36] (referred to as the lime network in this article) to classify limes into two categories, normal and defective, with an accuracy of 100%. The appearance of green plums is similar to green lemons. Python was used in this study to reproduce the CNN network with 18 layer designed by Jahanbakhshi, and green plum images were imported into the network for training to identify multiple defects of green plums. It can be seen from Table 4 that the average recognition rate of green plum defects by the green lemon network was 77.2%, but the recognition rate of rot defects reached 99%, and the green lemon network had a higher recognition rate of single defects. The green plum defect detection network is deeper than the green lemon network and can recognize more features. Therefore, the recognition rate of more complex scars and cracks is higher, so as to achieve a higher average recognition rate.

Resnet once won the first place in the classification task of the ImageNet competition [37]. It is a relatively new and efficient network that can solve the problem of gradient explosion and gradient disappearance caused by the deepening of the network. This article chooses the resnet18 network with similar layers to the green plum defect detection network for comparison. It can be seen from Table 4 that the average recognition rate of green plum defects on the resnet18 network is 90.18%, and the recognition rate of rot defects is 94.1%. The selection of the number of neurons in the classification layer of the green plum defect detection network is better, which shows a better advantage in the detection of green plum defect.

In order to evaluate the accuracy of the network model’s classification of green plum defects, the accuracy and recall criteria were used to evaluate the accuracy of the classification. The accuracy expresses the number of samples (TP) that the network model correctly predicts as positive (TP and FP). Taking the rot defect as an example, the green plums that were correctly identified as rot defects account for the proportion of all identified rot defects; the recall rate expresses the number of samples (TP) that the network model correctly predicted to be positive (the ratio of TP and FN), the ratio of correctly identified rot defects to actual rot defects. Since the number of samples between each defect is different, F1-Measure was used to establish a balance between precision and recall. The larger the value of F1-Measure, the better the classification of the model. The three parameters of precision, recall, and F1-Measure were used to judge the classification of the network model, as shown in Table 5 below.

It can be seen from the table above that the network model index evaluation result is that the green plum defect detection network is the best, followed by the resnet-18 network, the VGG network, and the worse is the lime network. The resnet-18 network is slightly better than VGG network in the detection of scar, crack, and rot. The lime network has obvious advantages in rot detection, but it is still difficult to identify multiple defects. The three index values of the green plum defect detection network in precision, recall, and F1-Measure are higher than the values of other networks. The green plum defect detection network has a value of 0.97 for the F1-Measure of the rot defect identification. The defect detection network model has the best effect on the identification of green plum rot defects, followed by the F1-Measure index value of normal green plums, which is 0.94, and the F1-Measure value of rain spot defects is 0.94.

The green plum defect detection network used a 3 × 3 small convolution kernel, which can identify richer features and increase the feature recognition rate. By using BN, the parameters were normalized to reduce the occurrence of excessive parameter changes due to different data distributions. The w-softmax loss function can help the network increase the gap between each category, reduce the gap between the same category, and improve the discrimination rate of features, thereby improving the classification accuracy. The SWA optimizer can achieve a good convergence effect, and the convergence speed is fast and stable. The results show that the green plum defect detection network can learn more green plum defect features, and the performance of green plum defect classification is better than other models.

## 5. Conclusions

In this study, based on the different defects on the green plum surface and multiple defects being difficult to correctly identify and classify, we introduced a convolution network to realize the multi-defect classification of green plums, and applied the SWA optimizer and w-softmax loss function to the green plum defect detection network. The network achieved an average recognition rate of 93.8% for the detection of green plum defects among which the recognition rate of green plum rot defects reached 99.25%, the recognition rate of normal green plums reached 95.65%, and the detection time of a single green plum image was 84.69 ms. The loss value of the green plum defect detection network and the VGG network during training was compared, and it was concluded that the loss value of the green plum defect detection network during training decreased faster, the loss value obtained was lower, and the model converged faster. The recognition rate of the green plum defect detection network was compared with the source VGG network and the lime network. The green plum defect detection network greatly improved the recognition of each defect. Finally, the performance evaluation of the three models verified the green plum defect detection network’s superiority compared with other network methods.

However, this study did not identify the green plum stems, which led to the stems of normal green plums being mistaken for defects. In the next experiment, the identification of green plum stems will be increased to further improve the classification accuracy of green plum defects. In this study, the recognition rate of scar and crack was low, which may be due to the small sample size of scar and crack data. In the next experiment, we will increase the sample size to further optimize the plum defect detection model.

In this study, a low-cost vision module composed of a camera and LEDs combined with a deep learning CNN network was used to detect and classify green plums on a static and single surface but failed to identify the entire green plum. In the next experiment, a comprehensive collection device for green plums can be designed to realize the online detection of green plum defects and provide technical support for automatic identification and sorting of green plum products for automated production.

## Figures and Tables

**Table 1:** Distribution of dataset.

	Rot	Spot	Scar	Crack	Normal
Original Dataset	390	400	140	80	230
Augmented Dataset	3900	4000	1400	800	2300
Training Dataset	3100	3200	1120	640	1840
Test Dataset	800	800	280	160	460

**Table 2:** Software and hardware environment configuration.

Name	Parameter
System	Windows 10 × 64
CPU	Inter Xeon W-2155@3.30 GHz
GPU	Nvidia GeForce GTX 1080 Ti(11G)
Environment configuration	PyCharm + Pytorch 1.2.0 + Python 3.7.7 Cuda 10.0 + cudnn 7.6 + tensorboardX 2.1.0
RAM	64 GB

**Table 3:** Results of green plum defect detection.

Method		Green Plum Defect Detection Network
Average precision of defect detection	Rot	99.25%
	Spot	93%
	Scar	84.29%
	Crack	78.13%
	Normal	95.65
Mean average precision		93.8%
Total Loss		0.02
Test time		84.69 ms

**Table 4:** Green plum defect detection results.

Method		VGG Network	Green Plum Defect Detection Network	Lime Network	Resnet-18 Network
Average precision of defect detection	Rot	89.38%	99.25%	99.0%	94.1%
	Spot	89.88%	93%	96.0%	86.6%
	Scar	78.93%	84.29%	50.0%	79.3%
	Crack	55.63%	78.13%	4.0%	62.5%
	Normal	89.88%	95.65%	8.7%	83.5%
Mean average precision		84%	93.8%	77.2%	90.18%
Test time		86.56 ms	84.69 ms	90.9 ms	92.34 ms

**Table 5:** Network evaluation.

		Green Plum Defect Detection Network	VGG Network	Lime Network	Resnet-18 Network
Recall	Scar	0.84	0.79	0.5	0.79
	Rot	0.99	0.89	0.99	0.94
	Normal	0.96	0.94	0.087	0.83
	Crack	0.78	0.56	0.04	0.63
	Spot	0.93	0.90	0.96	0.87
Precision	Scar	0.86	0.64	0.82	0.78
	Rot	0.94	0.92	0.88	0.91
	Normal	0.93	0.93	0.28	0.91
	Crack	0.93	0.74	0.055	0.67
	Spot	0.96	0.91	0.59	0.85
F1-Measure	Scar	0.85	0.70	0.62	0.79
	Rot	0.97	0.91	0.93	0.93
	Normal	0.94	0.93	0.13	0.87
	Crack	0.85	0.64	0.046	0.65
	Spot	0.94	0.91	0.73	0.86
